# The Anthocyanidins Malvidin and Cyanidin Alleviate Irinotecan-Triggered Intestinal Mucositis by Modulating Oxidative Stress and Cytokine Release

**DOI:** 10.3390/ijms262110747

**Published:** 2025-11-05

**Authors:** Giovana Filócomo Machado, Quélita Cristina Pereira, Felipe Leonardo Fagundes, Maycon Tavares Emílio-Silva, Vinícius Peixoto Rodrigues, Mariana de Almeida Patiño, Giulia Izzo Jorge, José Aires Pereira, Carlos Augusto Real Martinez, Clélia Akiko Hiruma-Lima, Raquel de Cássia dos Santos

**Affiliations:** 1 Post Graduate Program in Health Sciences, Laboratory of Natural Products, São Francisco University, Bragança Paulista 12916-900, São Paulo, Brazil; giovanafmachado2013@gmail.com (G.F.M.); quelitapereirapa@gmail.com (Q.C.P.); felipel_fagundes@hotmail.com (F.L.F.); 2 Department of Structural and Functional Biology, Physiology Sector, Institute of Bioscience, São Paulo State University (UNESP), Botucatu 18618-689, São Paulo, Brazil; maycon.silva@unesp.br (M.T.E.-S.); peixoto.rodrigues@unesp.br (V.P.R.); clelia.hiruma@unesp.br (C.A.H.-L.); 3 Post Graduate Program in Health Sciences, Laboratory of Medical Investigation, São Francisco Medical School, Bragança Paulista 12916-900, São Paulo, Brazil; mariana.patino@mail.usf.edu.br (M.d.A.P.); giuliaizzoj@gmail.com (G.I.J.); jose.pereira@usf.edu.br (J.A.P.); carlos.martinez@usf.edu.br (C.A.R.M.)

**Keywords:** anthocyanidin, chemotherapy, inflammation, intestinal mucositis, oxidative stress

## Abstract

Chemotherapy with irinotecan (CPT-11) induces intestinal mucositis via oxidative stress and NF-κB-driven cytokine amplification. We investigated the protective effects of the anthocyanidins cyanidin and malvidin (5 mg/kg) in a murine CPT-11 mucositis model. Both compounds increased duodenal glutathione level (GSH) and reduced lipid peroxidation (MDA), with distinct antioxidant profiles: malvidin enhanced catalase (CAT) activity, while cyanidin elevated superoxide dismutase (SOD). In the colon, cyanidin lowered MDA, whereas other oxidative and inflammatory markers remained largely unchanged. Malvidin significantly reduced IL-1β and IL-17 in both intestinal segments; cyanidin selectively decreased IL-6 in the colon, and this reduction was also observed for malvidin treatment. Gene expression analysis revealed broad transcriptional suppression in the duodenum for both compounds (*Nrf2*, *NF-κB*, *TNF-α*, *IL-1β*, *IL-6*, *IL-17*, *IL-10*), while colonic effects were more limited (suppression in *IL-6* for both compounds). Despite these biochemical and transcriptional improvements—which were more pronounced with malvidin—neither compound prevented CPT-11-induced weight loss or colonic histopathology, indicating that redox and cytokine modulation alone are insufficient to restore mucosal integrity. Overall, malvidin demonstrated a more significant modulation in the antioxidant response in the duodenum, with anti-inflammatory activity in both segments, while cyanidin showed targeted modulation of oxidative stress. These findings position both anthocyanidins as complementary agents with distinct mechanistic profiles, warranting further investigation into dose–response, pharmacokinetics, NRF2 protein dynamics, and barrier-repair strategies. Early-phase clinical evaluation is recommended to assess their potential as adjunctive therapies for chemotherapy-induced intestinal mucositis.

## 1. Introduction

Mucositis—ulcerative, inflammatory lesions of the oral and gastrointestinal mucosa—is a frequent, costly side effect of chemotherapy and radiotherapy. It drives bacteremia, malnutrition, higher analgesic use, prolonged hospital stays and often forces chemotherapy dose reductions that worsen patient survival and markedly increases healthcare use [1,2,3,4]. This disease often implies reducing or postponing chemotherapy, which contributes to increased mortality in patients with cancer. Depending on the chemotherapeutic regimen, the clinical incidence of intestinal mucositis (IM) may reach 40–76% [5] and can affect treatment intensity, duration, and effectiveness in approximately 50% of cases [6]. Since mucositis manifests systemically—accompanied by oral lesions, emesis, anorexia, abdominal pain, bleeding, fatigue, dehydration, diarrhea, and infections with potentially fatal complications—its study in humans remains particularly challenging [7]. Among interventions aimed at mitigating irinotecan-induced IM, natural compounds have garnered attention. For example, berberine attenuates mucosal injury by inhibiting bacterial β-glucuronidase activity without diminishing irinotecan’s antitumor efficacy [8], and glucagon-like peptide-2 analogues promote mucosal healing and reduce symptom severity [9]. Likewise, the flavonoid luteolin exerts PPARγ-dependent protection in human intestinal cells exposed to irinotecan [10], illustrating the diverse therapeutic strategies under exploration.

CPT-11, a quinolone alkaloid and semi-synthetic camptothecin derivative, functions as a prodrug that is converted by carboxylesterase into its active metabolite SN-38, predominantly in the liver and to a lesser extent in the gastrointestinal tract [11]. As a selective topoisomerase I inhibitor, SN-38 prevents DNA strand re-ligation, causing DNA breaks and inhibiting cell division [12]. Moreover, enterocyte-mediated reuptake of SN-38 maintains mucosal bioavailability at low concentrations and drives intestinal mucosal injury through continuous epithelial turnover and prolonged exposure, which, in turn, trigger pro-inflammatory signaling cascades [13]. Despite its well-characterized mechanism of action, irinotecan remains a cornerstone in the treatment of colorectal cancer [14], ovarian cancer [15], and Hodgkin’s lymphoma [16].

Its adverse effects—chiefly severe diarrhea, cholinergic syndrome, neutropenia, nausea, vomiting, alopecia, and asthenia—affect up to 80% of patients [17,18], with rodent models replicating significant diarrhea, villus atrophy, crypt loss, and increased intestinal cytokine synthesis (TNF-α and IL-1β) [19]. Additionally, alterations in gut microbiota, particularly an increase in β-glucuronidase-producing bacteria, exacerbate IM and elevate the risk of systemic infections [20,21]. Collectively, these findings underscore the urgent need for novel therapeutic approaches to mitigate irinotecan-induced mucosal damage and improve patient outcomes [22,23].

Considering these challenges and the growing interest in natural compounds with antioxidant and anti-inflammatory properties, anthocyanidins have emerged as promising candidates for IM prevention and treatment. These polyphenolic pigments—most notably cyanidin, malvidin, delphinidin, peonidin, petunidin, and pelargonidin—are abundant in fruits and vegetables and differ by their substituent patterns, including sugar and organic acid moieties [24]. According to Nurse’s Health Study II, the daily consumption of anthocyanidins is approximately 2–35 mg·kg-1 in women [25]. A single serving of berries can provide 100–200 mg of anthocyanidins, which is 5–10 times more than the amount found in other flavanols [26].

Cyanidin is the most common dietary anthocyanidin and its glycoside form is widely distributed in nature and malvidin, a structurally related anthocyanidin, is present in grapes and abundant in wine, where both its glycoside and aglycone forms can be found. A variety of pharmacological effects of cyanidin and malvidin have been reported in the literature, including the prevention of cardiovascular diseases, cancer, and diabetes, as well as anti-inflammatory and antioxidant activities [27,28,29,30], reducing gastric lesions in males and females [31], and malvidin repairing the gastric and duodenal mucosa in rodents [29]. Although few studies have directly examined their protective effects against CPT-11–induced mucositis, dietary red cabbage rich in anthocyanins markedly attenuated intestinal injury in irinotecan-treated rats [32], and anthocyanins were shown to reduce irinotecan’s genotoxicity by safeguarding DNA integrity [33]. However, the precise molecular mechanisms by which cyanidin and malvidin exert these benefits in IM remain unclear. Therefore, this study investigates the preventive and therapeutic potential of cyanidin and malvidin in a murine model of irinotecan-induced intestinal mucositis, with the aim of characterizing their mechanisms of action.

## 2. Results

### 2.1. Analysis and Monitoring of Survival and Body Weight Changes in Mice with Irinotecan-Induced Intestinal Mucositis

**Figure 1:**
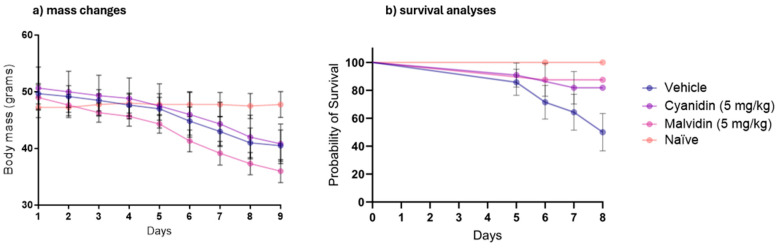
Effects of cyanidin and malvidin on body weight and survival in CPT-11-induced intestinal mucositis in mice. (**a**) Body weight changes over the course of chemotherapy. Values are mean ± SEM. Statistical differences were assessed by one-way ANOVA with Dunnett’s post hoc test. (**b**) Kaplan–Meier survival curves for the same treatment groups. Survival differences were evaluated using the log-rank (Mantel–Cox) test. Statistical differences versus vehicle-treated controls were determined by one-way ANOVA followed by Dunnett’s post hoc test. n = 5–8 per group.

Figure 1a presents the evolution of body mass of animals subjected to intestinal mucositis, and Figure 1b shows the survival analysis of the animals. No statistically significant differences were observed between the vehicle control and malvidin or cyanidin treatment groups. This indicates that the anthocyanidin treatments did not mitigate CPT-11-induced weight loss (Figure 1a) nor significantly altered the probability of survival in this animal model (Figure 1b).

### 2.2. Analysis of Antioxidant Potential of Anthocyanidins in CPT-11 Chemotherapy-Induced Mucositis

**Figure 2:**
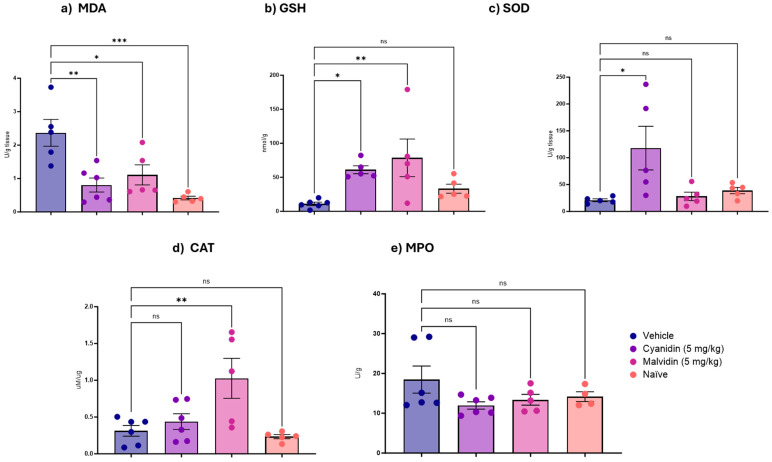
Effects of Malvidin and Cyanidin on oxidative stress (GSH, MDA, SOD, CAT) and inflammation (MPO) markers in the duodenum of mice with CPT-11-Induced Intestinal Mucositis. (**a**) Malondialdehyde levels, (**b**) reduced glutathione levels, (**c**) superoxide dismutase activity, (**d**) catalase activity, and (**e**) myeloperoxidase activity. Data are mean ± SEM (n = 5–6 per group). Statistical differences versus vehicle-treated controls were determined by one-way ANOVA with Dunnett’s post hoc test. * *p* < 0.05, ** *p* < 0.01, *** *p* < 0.001; ns, not significant.

**Figure 3:**
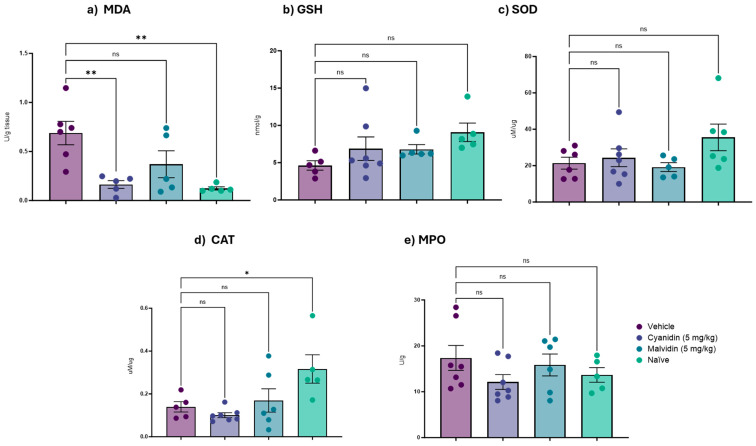
Effects of Malvidin and Cyanidin on oxidative stress (GSH, MDA, SOD, CAT) and inflammation (MPO) markers in the colon of mice with CPT-11-Induced Intestinal Mucositis. (**a**) Malondialdehyde levels, (**b**) reduced glutathione levels, (**c**) superoxide dismutase activity, (**d**) catalase activity, and (**e**) myeloperoxidase activity. Data are mean ± SEM (n = 5–6 per group). Statistical differences versus vehicle-treated controls were determined by one-way ANOVA with Dunnett’s post hoc test. * *p* < 0.05, ** *p* < 0.01; ns, not significant.

Chemotherapeutic agents generate oxidative stress through reactive oxygen species (ROS), which mainly damage DNA. In this context, we evaluated the antioxidant mechanisms of anthocyanidins. In the duodenum (Figure 2), GSH levels were significantly increased by both cyanidin and malvidin, and both treatments produced a reduction in MDA, indicating lowered lipid peroxidation. The antioxidant responses were compound-specific: malvidin increased CAT activity, whereas cyanidin increased SOD activity. Neither did anthocyanidin produce a meaningful decrease in myeloperoxidase (MPO) activity. In the colon (Figure 3), the only significant biochemical change detected was a reduction in MDA after cyanidin treatment; other measured antioxidant and inflammation markers in this tissue remained unchanged.

### 2.3. Quantification of Cytokines Involved in the Inflammatory Response

**Figure 4:**
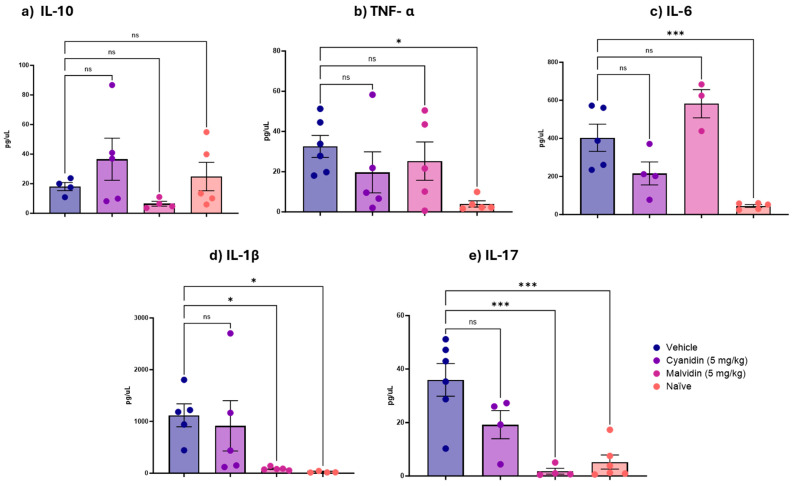
Duodenal cytokine levels in mice with irinotecan-induced mucositis following treatment with cyanidin or malvidin (5 mg/kg). Panels illustrate the following: (**a**) IL-10, (**b**) TNF-α, (**c**) IL-6, (**d**) IL-1β, and (**e**) IL-17. Data are mean ± SEM (n = 4–5 mice per group). Statistical differences versus vehicle controls were determined by one-way ANOVA followed by Dunnett’s post hoc test. * *p* < 0.05, *** *p* < 0.001; ns, not significant.

**Figure 5:**
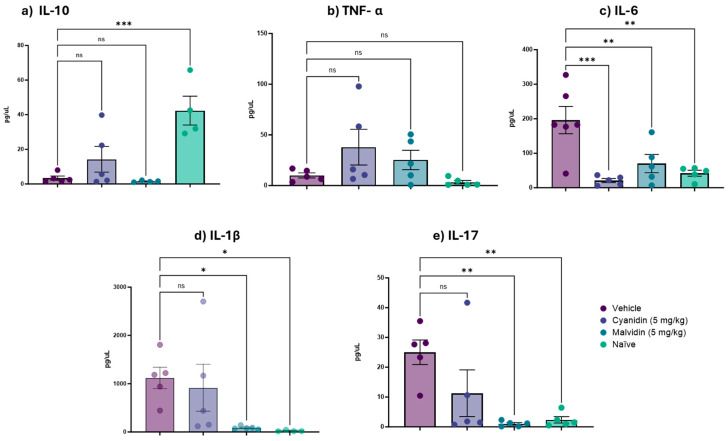
Effects of Cyanidin and Malvidin (5 mg/kg) on colonic cytokine levels in mice with irinotecan-induced intestinal mucositis. Panels illustrate the following: (**a**) IL-10, (**b**) TNF-α, (**c**) IL-6, (**d**) IL-1β, and (**e**) IL-17. Data are mean ± SEM (n = 4–5 mice per group). Statistical differences versus vehicle controls were determined by one-way ANOVA followed by Dunnett’s post hoc test. * *p* < 0.05, ** *p* < 0.01, *** *p* < 0.001; ns, not significant.

Figure 4 shows the levels of proinflammatory cytokines quantified in the duodenal segment following treatment with cyanidin and malvidin in the irinotecan-induced mucositis model. Malvidin administration resulted in decreased levels of IL-1β and interleukin-17 (IL-17), as well as a decrease in IL-6 in the duodenum. In the colonic segment (Figure 5), treatment with both malvidin and cyanidin produced a statistically significant decrease in IL-6. Malvidin further lowered IL-1β and IL-17 levels in the colon, replicating the cytokine-suppressive profile observed in the duodenum.

### 2.4. Gene Expression Analysis of Inflammatory and Antioxidant Markers

**Figure 6:**
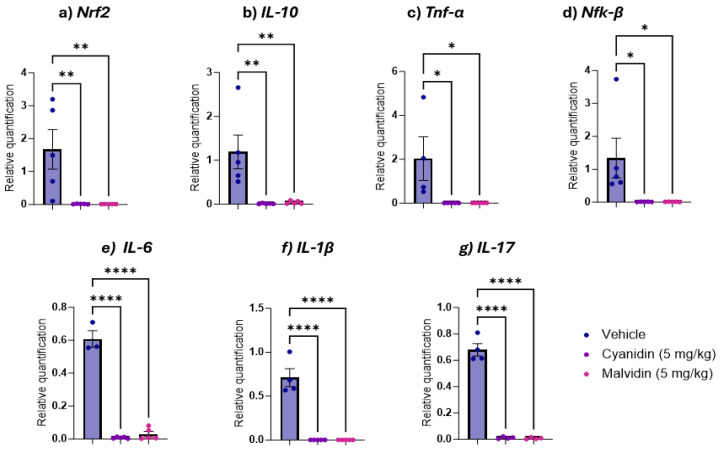
Duodenal gene expression of antioxidant and inflammatory markers in mice with CPT-11-induced intestinal mucositis treated with Malvidin or Cyanidin (5 mg/kg). (**a**) *Nrf2* (nuclear factor erythroid 2-related factor 2), (**b**) *IL-10* (interleukin 10), (**c**) *TNF-α* (tumor necrosis factor alpha), (**d**) *NF-κB* (nuclear factor kappa B), (**e**) *IL-6* (interleukin 6), (**f**) *IL-1β* (interleukin 1 beta) and (**g**) *IL-17* (interleukin 17). mRNA levels were quantified by qRT-PCR and normalized to GAPDH. Data are presented as mean ± SEM (n = 3–5 mice per group). Statistical differences versus vehicle controls were determined by one-way ANOVA followed by Dunnett’s multiple comparisons test. * *p* < 0.05, ** *p* < 0.01, **** *p* < 0.0001.

**Figure 7:**
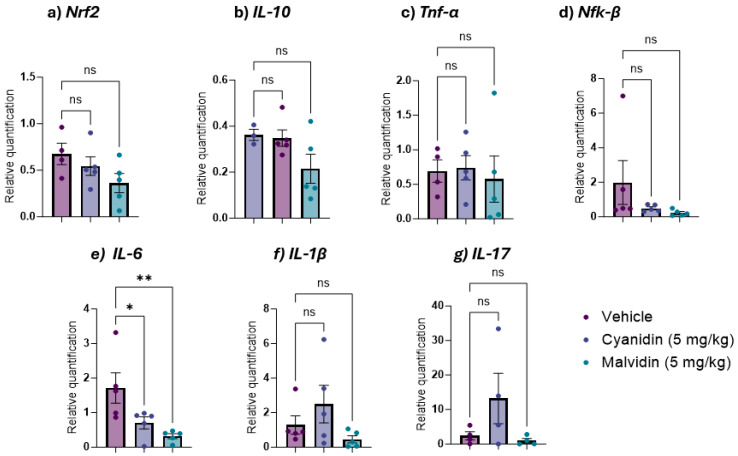
Colonic gene expression of antioxidant and inflammatory markers in mice with CPT-11-induced mucositis treated with malvidin or cyanidin (5 mg/kg). (**a**) *Nrf2* (nuclear factor erythroid 2-related factor 2), (**b**) *IL-10* (interleukin 10), (**c**) *TNF-α* (tumor necrosis factor alpha), (**d**) *NF-κB* (nuclear factor kappa B), (**e**) *IL-6* (interleukin 6), (**f**) *IL-1β* (interleukin 1 beta) and (**g**) *IL-17* (interleukin 17). mRNA levels were quantified by qRT-PCR and normalized to 18S. Data are mean ± SEM (n = 3–5 mice per group). Statistical differences versus vehicle controls were determined by one-way ANOVA followed by Dunnett’s post hoc test. * *p* < 0.05, ** *p* < 0.01; ns, not significant.

The gene expression analysis of inflammatory cytokines revealed distinct profiles between the duodenal and colonic portions in the CPT-11-induced mucositis model. Treatment with both anthocyanidins produced a significant downregulation of every gene assessed (Figure 6): *Nrf2*, *IL-10*, *Tnf-α*, *Nf-kβ*, *IL-6*, *IL-1β* and *IL-17*. In the colon, this transcriptional suppression was more selective: both malvidin and cyanidin significantly lowered *IL-6* expression (Figure 7).

### 2.5. Histopathological Analyses

**Figure 8:**
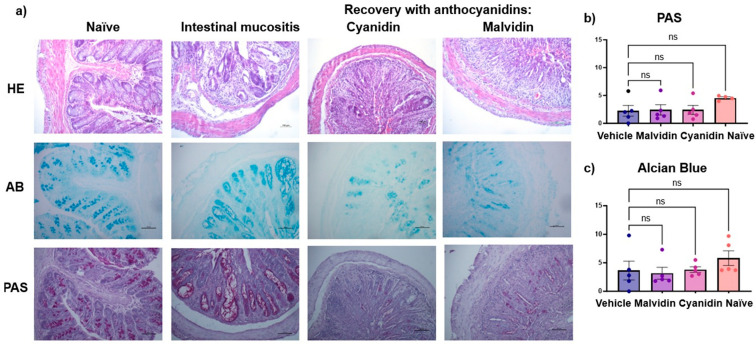
Histopathological assessment of colonic tissue in mice with CPT-11-induced mucositis treated with malvidin or cyanidin (5 mg/kg) or vehicle (0.9% saline). (**a**) Representative colon sections stained with hematoxylin–eosin (H&E), alcian blue (AB) for acidic mucins, and periodic acid–Schiff (PAS) for neutral mucins (200×; scale bar = 100 μm). (**b**) PAS-positive mucin area, expressed as percentage per field. (**c**) AB-positive mucin area, expressed as percentage per field. Images were analyzed in NIS-Elements software 2.3: RGB histograms quantified pixel intensity, and results represent the average of three randomly selected fields per sample. Data are mean ± SEM (n = 4–6 mice per group). One-way ANOVA with Dunnett’s post hoc test; ns, not significant.

Histopathological examination revealed inflammation in all treated groups exept for naïve group, with varying severity and tissue damage. Active hyperemia was present in most samples, reflecting the physiological response to inflammation. In vehicle-treated mice, inflammation ranged from mild to moderate. Mice treated with cyanidin or malvidin also showed inflammatory lesions—marked by epithelial disruption and glandular atrophy—indicating that these anthocyanidins did not preserve acidic or neutral mucins and that irinotecan’s damaging effects persisted despite treatment. Figure 8a displays representative colon sections from naïve, vehicle, cyanidin, and malvidin groups stained with hematoxylin–eosin, periodic acid–Schiff, and alcian blue. Figure 8b quantifies mucin content, confirming that neither anthocyanidin altered acid or neutral mucin levels relative to the vehicle controls.

## 3. Discussion

Intestinal mucositis (IM) is a common gastrointestinal condition in patients undergoing chemotherapy and can be initiated when chemotherapeutics trigger excessive reactive oxygen species (ROS) and activate NF-κB, followed by upregulation of pro-inflammatory cytokines (e.g., TNF-α, IL-1β), amplification of these signals, epithelial ulceration through apoptosis, and finally a spontaneous—but often incomplete—healing phase [1,23]. As expected, repeated CPT-11 dosing caused a pronounced decline in body weight and a significant mortality rate among vehicle-treated mice. Despite failing to prevent weight loss, malvidin—but not cyanidin—nearly halved the death rate, underscoring its capacity to improve survival under extreme mucosal injury without detectable organ toxicity. Following our macroscopic assessments, we proceeded to examine the mechanisms that drive antioxidant protection

Chemotherapy impairs endogenous antioxidant systems—SOD, CAT and —thereby exacerbating lipid peroxidation and barrier injury [23,34]. Enzymatic antioxidants, such as CAT and SOD, decompose hydrogen peroxide into water and oxygen without generating free radicals [35,36]. Both anthocyanidins restored redox homeostasis: malvidin and cyanidin increased duodenal GSH, CAT activities and reduced MDA levels (*p* < 0.05). This action has been observed in other studies with these molecules [28,37]. These findings align with the role of enzymatic antioxidants in decomposing hydrogen peroxide into innocuous products, thus interrupting deleterious ROS cascades [34,38], corroborating results observed in other studies [29,39]. Antioxidants mitigate the deleterious effects of ROS through direct elimination, limiting oxidant propagation, and inhibiting oxidative stress [40]. Malvidin efficiently modulated CAT activity in models of peptic ulcer prevention and repair, highlighting the importance of this pathway in the pharmacological activity of anthocyanidins [41]. Catalase activity is also related to maintaining redox balance in cells and reducing the generation of reactive oxygen species [42,43]. Even when the agent causing intestinal damage was CPT-11, cyanidin significantly increased GSH activity. Importantly, malvidin and cyanidin restored redox homeostasis within the duodenal mucosa. Malvidin robustly increased glutathione (GSH) and catalase (CAT) activities while reducing lipid peroxidation (MDA). Cyanidin likewise enhanced GSH levels and lowered MDA levels, reflecting direct ROS scavenging and enzyme induction [44]. These findings align with the known capacity of anthocyanidins to boost endogenous antioxidant defenses by up-regulating SOD, CAT, and phase II detoxifying enzymes [45].

Neutrophil-derived myeloperoxidase (MPO) activity, a marker of inflammatory cell infiltration, was elevated by CPT-11 and significantly suppressed by malvidin in both duodenum and colon [46], indicating reduced mucosal inflammation [23]. Cyanidin exerted a more modest MPO-lowering effect, consistent with its intermediate antioxidant potency.

To dissect the inflammatory milieu, we quantified key cytokines. IL-1β, a master regulator of mucosal inflammation that synergizes with TNF-α to drive epithelial apoptosis, was robustly downregulated by malvidin (*p* < 0.01), as were TNF-α and IL-17 in the duodenum and IL-6/IL-17 in the colon (*p* < 0.05). These multifunctional inflammatory cytokines play vital roles in host defense, acute-phase reactions, immune responses, hematopoiesis, and cell survival. It is rapidly induced during acute inflammatory reactions [47] and is essential for maintaining intestinal homeostasis by interacting with the immune epithelial system and supporting mucosal integrity [48]. In the present study, it was possible to observe a decrease in inflammatory processes in the duodenum, signaled by the TNF-α marker in the intestinal mucositis induction protocol with CPT-11 [11], when malvidin was administered to the animals. Therefore, through pro-and anti-inflammatory markers, our results demonstrated that although chemotherapeutic drugs induced inflammatory processes, anthocyanidins were able to prevent or reduce these processes, represented by a decrease in inflammatory cytokines. Cyanidin significantly decreased IL-6 and TNF-α in both segments (*p* < 0.05). These data corroborate the multistage pathogenesis of IM—where early ROS and NF-κB activation beget a cytokine storm—and underscore anthocyanidins’ ability to quell both oxidative and cytokine-mediated damage [23,34].

Anthocyanidins markedly attenuated the inflammatory cascade triggered by irinotecan (CPT-11) in our murine mucositis model. Both compounds significantly reduced IL-1β release in the duodenum and colon, consistent with inhibition of NF-κB–mediated transcription of inflammatory genes [49]. Continuing with these analyses, the next most abundant cytokine was IL-6. Our results showed that both anthocyanidins reduced IL-6 levels in at least one intestinal region during mucositis induction, demonstrating their anti-inflammatory properties. IL-6, a key driver of mucosal injury and neutrophil recruitment, was also suppressed by malvidin in both intestinal segments and by cyanidin in the colon, underscoring their ability to interrupt early amplification of inflammation [37].

Despite their potent effects on pro-inflammatory mediators, neither anthocyanidin altered IL-10 levels. As a key anti-inflammatory cytokine, IL-10 restrains Th1/Th17 responses and promotes M2 macrophage polarization, thereby limiting excessive immune activation and tissue damage [47]. Its unchanged levels suggest that malvidin and cyanidin primarily act upstream by modulating NF-κB–dependent signaling pathways, rather than broadly enhancing regulatory cytokine expression [50]. In contrast, IL-17—central to neutrophil recruitment, barrier disruption and recruitment and survival of neutrophils, resulting from antigenic stimulation of dendritic cells [47,51]—was significantly suppressed by malvidin, aligning with its documented inhibition of Th17-differentiation and neutrophil chemotaxis [28].

In addition, the involvement of gene expressions of the same markers involved in the inflammatory process (*IL-1β*, *IL-6*, *IL-17*, *TNF-α*, *IL-10*), *Nrf2*, and *NF-kβ* expression were examined. The results observed in the relative expression of malvidin in the irinotecan protocol seemed to be positively correlated with those verified by the quantification of cytokines, indicating that the regulation of expression interacts with the amount of release of these cytokines. In contrast to what was observed for IL-6 in the quantification of the cytokine, when proceeding with the analysis of gene expression, an inverse response was observed when analyzing gene expression, with a significant increase in the expression of this cytokine. Although the increase in gene expression suggests non-participation of this pathway, the cytokines detected in intestinal tissues were reduced, suggesting that anthocyanidins can modulate this release, indicating an important mechanism of action that requires further investigation. Together, these data position malvidin and cyanidin as dual-action agents that reinforce redox homeostasis while dampening key cytokine networks in chemotherapy-induced intestinal mucositis.

Chemotherapy-induced intestinal damage involves mucosal inflammation and histological disruption, resulting from stem cell apoptosis and impaired epithelial renewal. These changes alter secretion, absorption, and mucus production, compromising the protective barrier. Mucins modulate epithelial proliferation and resistance to apoptosis and influence the severity of mucositis [23,52]. Mucin levels in the colon remained unchanged in CPT-11-induced mucositis, suggesting that anthocyanidins exert their pharmacological effects through pathways independent of mucin modulation.

Our data indicates a clear compartmentalization of anthocyanidin effects across the small and large intestine that can be explained by differences in local exposure, metabolic processing, and distinct modes of action. Orally administered polyphenols encounter the small intestinal epithelium at higher parent-compound concentrations and relatively limited microbial transformation, favoring early biochemical interactions with epithelial cells and resident immune populations. This higher early exposure plausibly underlies the duodenum’s pronounced response: robust increases in GSH and enzyme activities (SOD/CAT) together with broad downregulation of inflammatory and oxidative stress genes. In this setting anthocyanidins appear to operate through a dual, complementary mechanism—immediate antioxidant support plus upstream suppression of inflammatory transcriptional programs. Immediate antioxidant support is reflected by thiol replenishment and induction of peroxide-detoxifying enzymes, which interrupt ROS propagation and lower lipid peroxidation; transcriptional suppression of NF-κB and related cytokine genes then reduces the magnitude and persistence of the cytokine amplification loop that drives epithelial apoptosis and barrier failure.

By contrast, the colon is dominated by extensive microbial biotransformation of parent anthocyanidins into diverse metabolites that may have different potency, stability, or cellular targets. Reduced availability of the active parent molecules, the generation of metabolites with weaker transcriptional activity, and the colon’s distinct epithelial turnover and immune milieu together explain the attenuated and more selective colonic responses we observed: a clear reduction in lipid peroxidation (MDA) after cyanidin but only selective decreases in cytokines (notably IL-6, and malvidin-linked reductions in IL-1β/IL-17). This pattern is consistent with a predominately ROS-scavenging or metabolite-mediated action in the large intestine that does not produce the same breadth of NF-κB transcriptional remodeling seen in the duodenum.

Together, these observations support a model in which anthocyanidins act as site-adapted modulators: in the duodenum, they deliver both rapid biochemical antioxidant effects and transcriptional repression of inflammatory drivers, while in the colon, they primarily reduce oxidative damage through scavenging or metabolite activity with more limited effects on transcriptional networks. This compartmentalized pharmacology highlights the importance of measuring both biochemical endpoints and transcriptional/protein markers across intestinal segments, and it points to concrete next steps to confirm the causal chain from exposure to molecular effect to tissue protection.

The present study yielded significant findings; however, limitations must be acknowledged: (a) Single-dose design—using only one dose per compound precludes assessment of dose–response relationships and optimal therapeutic windows. (b) Rodent model translatability—murine intestinal physiology and drug metabolism differ from humans, creating a translational gap that may affect clinical efficacy predictions. (c) Lack of healthy tissue data—without examining anthocyanidin effects in non-pathological intestines, we cannot exclude baseline modulatory actions or off-target effects. In summary, our preclinical data identify cyanidin and malvidin as strong candidates for managing CPT-11-induced intestinal mucositis by simultaneously bolstering antioxidant defenses (↑GSH, SOD, CAT; ↓MDA) and suppressing key pro-inflammatory mediators (IL-1β, IL-6, IL-17, TNF-α, NF-κB). In the absence of any approved therapies for intestinal chemotherapy-induced mucositis, these results are particularly encouraging.

## 4. Material and Methods

### 4.1. Animals

Animal studies were conducted following the ARRIVE GUIDELINES [53]. Male Swiss mice (*Mus musculus*), aged 10–14 weeks and weighing approximately 50 g were provided by the Multidisciplinary Center for Biological Investigation in the Science of Laboratory Animals (Unicamp, SP, Brazil) after approval by the Ethics Committee in Animal Use of Universidad São Francisco (protocol number 001.12.2017, 005.03.2020). The animals were maintained under standard conditions (12 h light/dark cycle and room temperature of 22 ± 2 °C), fed a Presence^®^ diet, and had free access to water. The Presence^®^ diet is a complete and extruded food formulated with balanced grains, bran, vitamins, and minerals to meet all the nutritional needs of laboratory rodents. All experiments were conducted according to international and Brazilian standards of animal welfare. The animals were randomly divided into groups, and all experiments were performed in a blind manner.

### 4.2. Experimental Design: Induction of Intestinal Mucositis

**Figure 9:**
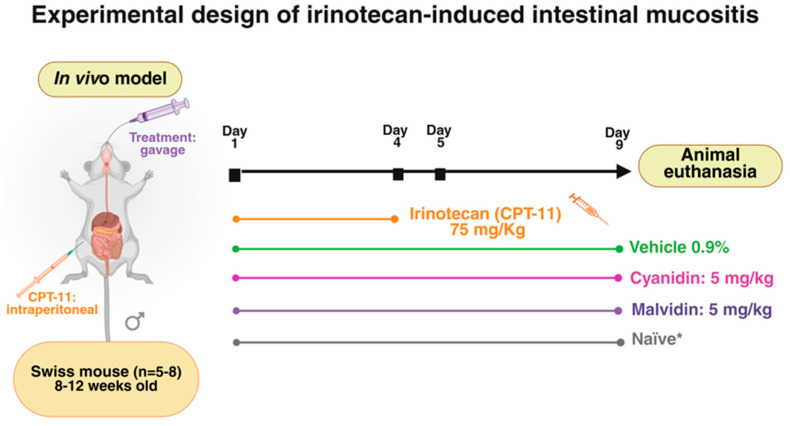
Experimental protocol for irinotecan-induced intestinal mucositis in mice. Mice were randomized into four groups (n = 5–8 per group): Naïve (*)—no irinotecan or anthocyanidin treatment (physiological baseline), Vehicle control—0.9% saline orally, Cyanidin—5 mg/kg orally, Malvidin—5 mg/kg orally. Irinotecan (CPT-11, 75 mg/kg) was administered intraperitoneally according to the schedule shown. Oral treatments (cyanidin, malvidin, or saline) were administered coincident with the beginning of the first CPT-11 dose and continued until the end of the study. Endpoint analyses (body weight, histology, biochemistry, gene expression) were performed on day 9. * Naïve mice were not subjected to any treatment or handling, serving as a reference for normal physiological parameters. Created in BioRender. Pereira, Q. (2025) https://BioRender.com/nk1pf8f.

The mice were randomly divided into four groups: the naïve group (n = 5), which were kept under normal living conditions as a control without receiving any substance; and the treated groups, which received malvidin or cyanidin (5 mg/kg), or vehicle (0.9% saline) (starting with 8 animals per group). The doses were selected from previous studies with the molecules [29,31]. The body weight of the mice was monitored daily throughout the experimental protocol. Intestinal mucositis was induced by intraperitoneal administration of CPT-11 (75 mg/kg) during the first four days of the experiment. Concomitantly, malvidin (5 mg/kg), cyanidin (5 mg/kg), or vehicle (0.9% saline) were administered orally until day 8. At the end of the experiment, the animals were euthanized using lethal combination of ketamine (100 mg/mL) and xylazine (2%). The untreated group (naïve) was included in the comparative analysis (Figure 9).

### 4.3. Antioxidant Parameters

For the evaluation of antioxidant activity, activity of SOD and CAT, levels of MDA and GSH were analyzed in intestinal tissue samples. Colon and duodenum samples were homogenized in extraction buffer (1:4, *v*/*v*) containing 1% protease inhibitor [54], using a Polytron homogenizer for approximately 25 s. The homogenates were then centrifuged at 19,000 rpm for 15 min, and the supernatant was collected for enzymatic analysis. Total protein concentration was determined using the biuret method with a Protal kit (Laborclin, Pinhais, Paraná, Brazil). SOD activity was assessed based on the reaction with hypoxanthine, xanthine oxidase, and NBT, with absorbance read at 560 nm [36]. GSH levels were quantified using DTNB, with measurements taken at 414 nm. CAT activity was determined based on the decomposition of H_2_O_2_ and the formation of formaldehyde, read at 540 nm [41]. Malondialdehyde levels were measured by tiobarbituric acid reactive substances (TBARS) method, in which samples were incubated with thiobarbituric acid at 95 °C, and absorbance was measured at 532 nm. All results were normalized to total protein concentration and quantified using standard curves specific to each assay. Myeloperoxidase (MPO) activity was measured at 540 nm in the presence of hydrogen peroxide and o-dianisidine [28]. Results were expressed as units per gram of tissue, as previously described.

Cytokine levels of tumor necrosis factor alpha (TNF-α), interleukin 1 beta (IL-1β), interleukin 6 (IL-6), interleukin-17 (IL-17), and interleukin 10 (IL-10) were measured by enzyme-linked immunosorbent assay (ELISA) using mouse cytokine ELISA kits from R&D Systems (Minneapolis, MS, USA), according to the manufacturer’s protocol.

### 4.4. Quantitative qPCR Analyses

Duodenal and colon tissue samples were maintained in RNA later solution (Invitrogen, CA, USA) were extracted using the Trizol Reagent. For qRT-PCR, total RNA was reverse-transcribed using the High-Capacity cDNA Reverse Transcription Kit (Applied Biosystems, Foster City, CA, USA). The synthesized cDNAs was amplified using the SYBR Green PCR Master Mix (Qiagen, Hilden, Germany). The reaction was performed using a 7300 nm Detection System. The relative amount of the target gene was calculated using the -2∆∆CT method with 18S or *Gadph* as an endogenous control. The genes analyzed are in Table 1. Primer pairs were designed based on the validated FASTA sequence using the NCBI database and employing Primer3 software with the following parameters: primer length, 20–22 bases; melting temperature, 60 °C; amplicon size, 95–110 base pairs [36,55].

### 4.5. Histology

Histopathological analysis was performed to verify the presence of inflammatory processes in the colon. The techniques used included (HE) Hematoxylin and Eosin (PAS), Periodic Acid Schiff (PAS), and Alcian Blue (AB). HE stains the nucleus purple or blue and cytoplasm pink, allowing verification of the general morphology and processes of inflammation and necrosis. AB stains acidic mucins and secretions blue/turquoise. Finally, PAS stains neutral mucins and basement membrane red. The objective of using them together was to complement these techniques and obtain a more accurate diagnosis. Slide analysis was conducted using a Nikon Eclipse DS-50 optical microscope at 100× magnification. Images were captured with a DS-Fi-50 digital camera and processed using the NIS-Elements 2.3 software (Nikon Instruments Inc. 1300 Walt Whitman Road Melville, NY 11747-3064, USA) for image analysis. Using histograms in the RGB system, the program calculated the intensity of the colors in pixels and expressed the results as percentage per field (%/field). For each segment, the average of the three analyzed fields was considered.

### 4.6. Statistical Analysis

Each set was analyzed by an investigator blinded to the experimental groups. The Kolmogorov–Smirnov test was used to verify data normality. The data are expressed as mean ± SEM, and an analysis of variance (ANOVA) was applied to verify the difference between the means followed by Dunnett’s test. All datasets were analyzed for the presence of outliers using the Rout’s test. Statistical analyses were performed using the GraphPad Prism software (version 10.0; San Diego, CA, USA). Statistical significance was set by 0.05.

### 4.7. Material

Cyanidin hydrochloride and malvidin hydrochloride (>98% purity) were obtained from the Cayman Chemical Company (Ann Arbor, MI, USA). Tri Reagent, HTAB, Purpald, Xanthine Oxidase from microorganisms, Orto-Dianisidine Hydrochloride, Sodium Hydroxide, Potassium Hydroxide, Potassium Dihydrogen Phosphate, Sodium Orthovanadate, Sodium Pyrophosfate, Sodium Fluoride, Triton X-100 and Reduced Glutathione were obtained from Sigma Aldrich (Saint Louis, MO, USA). Xylazine Hydrochloride and Ketamine Hydrochloride were obtained from the Sespo Industry (Paulínea, São Paulo, Brazil). Iodine periodate, absolute ethanol, and hydrogen peroxide were obtained from Synth Chemistry (São Paulo, SP, Brazil). Methanol and hydrochloric acid were obtained from J. T. Backer (Xalostoc, Mexico). Hypoxanthine, nitroblue tetrazolium, and DTNB were purchased from Alfa Aesar (Tewksbury, MA, USA). EDTA, Tris, protease inhibitor cocktail, bovine serum albumin, and catalase were obtained from Thermo Fisher Scientific (Waltham, MA, USA). PMSF was purchased from Amresco (Solon, OH, USA), and Sodium Dodecyl Sulfate was purchased from Merck (Darmstadt, Germany).

## 5. Conclusions

Our study demonstrates that oral administration of cyanidin and malvidin (5 mg/kg) substantially attenuates CPT-11 (75 mg/kg)-induced intestinal mucositis in mice. Treated animals showed enhanced antioxidant defenses—significant increases in GSH, CAT, and SOD—and reduced lipid peroxidation (TBARS), especially with cyanidin. Both anthocyanidins also suppressed key pro-inflammatory cytokines (IL-1β, IL-6, IL-17) at protein and gene levels, modulating TNF-α, NF-κB pathways, and lowering MPO activity. Interestingly, IL-10 remained unchanged, suggesting a selective effect on pro-inflammatory cascades. Anthocyanidins cyanidin and malvidin provide segment-specific protection in CPT-11–induced intestinal mucositis by reinforcing antioxidant defenses and suppressing proinflammatory signaling. Future work should define optimal dosing, validate efficacy in higher-order models or patient-derived organoids, and explore early-phase clinical trials to translate these natural anthocyanidins into a viable supportive care strategy.

## Figures and Tables

**Table 1:** Primer sequence was used to investigate the influence of anthocyanidin treatment in the intestinal mucositis induced by CPT-11.

Gene	Sequence 5′-3′
*IL-17*	5′-CAGACTACCTCAACCGTTCCAC-3′
	5′-TCCAGCTTTCCCTCCGCATTAGA-3′
*GAPDH*	*5′-TCTCCACACCTATGGTGCAA-3′*
	5′-CAAGAAACAGGGGAGCTGAG-3′
*Nf-kB*	5′-CACAGAGGGCAAGGAAGAAG-3′
	5′-CCTGCCTCTCCTAACACTGC-3′
*18S*	5′-AAACGGCTACACCTCCAAG-3′
	5′-AAACGGCTACCACATCCAAG-3′
*IL-1β*	5′-CCCAAGCAATACCCAAAGAA-3′
	5′-TACCAGTTGGGGAACTCTGC-3′
*Tnf-α*	5′-TAGCCAGGAGGGAGAACAGA-3′
	5′-TTTCTGGAGGGAGATGTGG-3′
*IL-6*	5′-TCTCTGGGAAATCGTGGAA-3′
	5′-TTCTGCAAGTGCATCATCG-3′
*IL-10*	5′-AAAAGGTGCCACCCTGAAGA-3′
	5′-GATGTGGTGGGACCAACCTT-3′
*Nrf2*	5′-CCCAGGGTTTGAAAAGTGAA-3′
	5′-GCTGGAAAGTGAAGGCAGTC-3′
	5′-GTGCAACAGAAGAGCCATCA-3′

## Data Availability

The data supporting this study are included in the article, and if further information is required, the authors will make it available as requested.
